# Changes in structural and pigmentary colours in response to cold stress in *Polyommatus icarus* butterflies

**DOI:** 10.1038/s41598-017-01273-7

**Published:** 2017-04-25

**Authors:** Krisztián Kertész, Gábor Piszter, Zsolt Endre Horváth, Zsolt Bálint, László Péter Biró

**Affiliations:** 1 Institute of Technical Physics and Materials Science, Centre for Energy Research, Budapest, Hungary; 2 Hungarian Natural History Museum, Budapest, Hungary

## Abstract

While numerous papers have investigated the effects of thermal stress on the pigmentary colours of butterfly wings, such studies regarding structural colours are mostly lacking, despite the important role they play in sexual communication. To gain insight into the possible differences between the responses of the two kinds of colouration, we investigated the effects of prolonged cold stress (cooling at 5 °C for up to 62 days) on the pupae of *Polyommatus icarus* butterflies. The wing surfaces coloured by photonic crystal-type nanoarchitectures (dorsal) and by pigments (ventral) showed markedly different behaviours. The ventral wing surfaces exhibited stress responses proportional in magnitude to the duration of cooling and showed the same trend for all individuals, irrespective of their sex. On the dorsal wing surface of the males, with blue structural colouration, a smaller magnitude response was found with much more pronounced individual variations, possibly revealing hidden genetic variations. Despite the typical, pigmented brown colour of the dorsal wing surface of the females, all cooled females exhibited a certain degree of blue colouration. UV-VIS spectroscopy, optical microscopy, and scanning and transmission electron microscopy were used to evaluate the magnitude and character of the changes induced by the prolonged cold stress.

## Introduction

During the past decade, the colour patterns of insects have emerged as model systems for studying the interplay between development and evolution^1^. The complex patterns encountered on butterfly wings are more often generated by various pigments^1^ than by structural colours^2, 3^, but composite patterns, having both pigmentary and structural origins, also occur^4^. The structural colours of butterflies, generated by sophisticated nanoarchitectures that are able to manipulate light propagation^2, 5^, are frequently used in sexual communication and may be an indicator of mate quality^6^. For example, it has been shown experimentally that the mate choice of female *Bicyclus anynana* butterflies is influenced by the dorsal white and UV-reflective eyespot pupils of the male wings^7^.

Temperature may have important effects on the development of butterflies, causing males and females to reciprocally change their sexual roles depending on their larval rearing temperatures, as observed in *Bicyclus anynana* butterflies^8^. In other species, the eyespot colours in the wings may have both pigmentary and structural origins, and the two kinds of colours are influenced by rearing conditions in different ways^4, 9–12^. In an extraordinary field case of a species of lycaenid butterfly, *Zizeeria maha*, for which plastic phenotypes of wing colour patterns were revealed at the population level during the course of range expansion in Japan, it was confirmed by laboratory experiments that the outbreak of modified phenotypes in the recent range margin population was primed by the revelation of plastic phenotypes in response to temperature stress^13^. Otaki and Yamamoto also showed that the wing pattern modifications are species specific^14^. A comprehensive review of physiologically induced colour-pattern changes in response to tungstate or cold-shock treatments was recently published^15^.

Although the structural colours of butterflies have been extensively studied in recent years^16–22^, cases in which the structural colour is homogenous over the whole, or majority of the wing surface, have not been investigated under environmental stress, with the exception of a recent work on *Colias eurytheme* ^6, 23^. We were interested to observe the effects of low-temperature treatment (cold shock or cold stress, i.e., prolonged cooling) on the structural colours and to compare these alterations with those of the pigmentary patterns. The mechanism of colour generation in structurally coloured butterfly scales is related to complex nanoarchitectures, composed mainly of chitin, which behave as photonic crystals^2^ and reflect light in certain wavelength ranges because of their characteristic nanostructure^5, 16, 21^. This is very different from the way in which colour is generated by pigments, which occurs through light absorption in the pigment molecules (chemical colour). Blue colour of structural origin is frequently used for sexual signalling and, as we have shown previously, is species specific^5^. An investigation of the biological variance of this colour in male *Polyommatus icarus* butterflies showed that while the amplitude of the blue reflectance maximum had a normal (Gaussian) distribution around the mean value, the spectral position of the maximum had a rectangular distribution (“box-like” profile), with a sharp drop at ±10 nm from the median value of 385 nm, indicating that the probability of the occurrence of individuals with outlier colours was very low^24^. Therefore, we believe that the structural blue colour of male *P. icarus* butterflies is more stable against stress-induced variation compared to the pigmentary pattern of the ventral wing surface used for the identification of this species. As a stress factor, we chose prolonged cooling of the pupae because this allowed us to also investigate the effects of the duration of the applied stress.

In the case of *Junonia orithya*, the onset of scale development occurs at approximately 30 hours after pupation, while the homogeneous black pigmentation process of the wing occurs at 150 hours post pupation^25^. The formation of the nanostructure and the pigmentation may be well separated in time, which underscores the importance of investigating the possible differences in the ways in which prolonged cold shock may affect the colours and patterns generated by nanoarchitectures and those produced by pigments.

We performed experiments with *Polyommatus icarus* larvae reared under laboratory conditions and subjected to cooling at 5 °C in a refrigerator immediately after pupation for durations ranging from 10 days to 62 days. After the cooling period, the pupae were allowed to mature and hatch at room temperature in the laboratory. Modifications of the structural and pigmentary colours were investigated in detail by UV-VIS spectroscopy and optical and electron microscopy, as well as a quantitative evaluation of the modifications of the pigmented patterns on the ventral wing surface. Data of wild exemplars^24^ were used for comparison.

**Figure 1:**
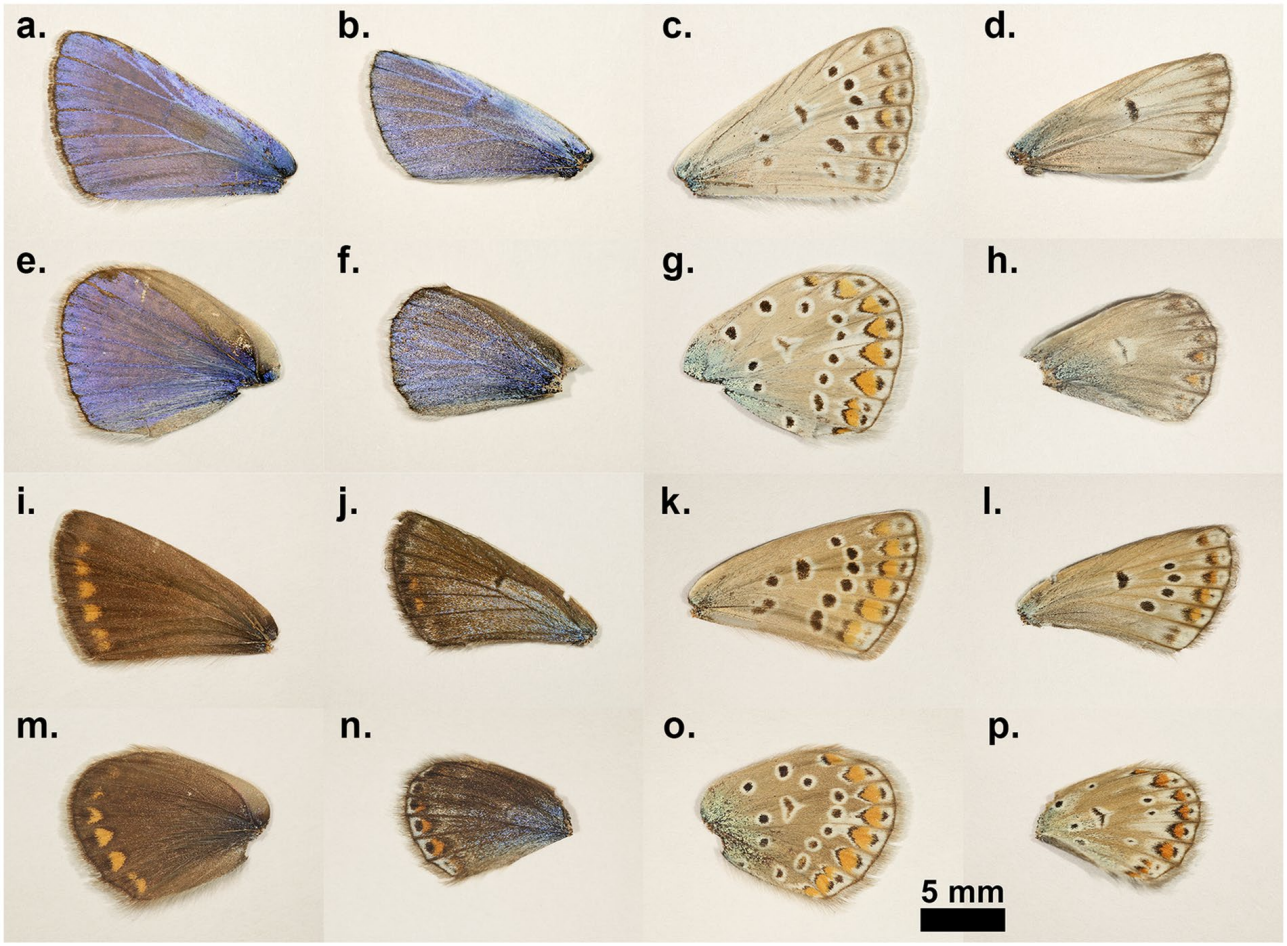
Wings of wild butterflies and of butterflies eclosed form pupae subjected to prolonged cooling at 5 °C in dark. (**a**) Dorsal forewing of a wild male; (**b**) dorsal forewing of a male emerged from a pupa cooled for 62 days; (**c**) ventral forewing of a wild male; (**d**) ventral forewing of a male eclosed form a pupa cooled for 62 days; (**e**) dorsal hindwing of a wild male; (**f**) dorsal hindwing of a male emerged from a pupa cooled for 62 days; (**g**) ventral hindwing of a wild male; (**h**) ventral hindwing of a male eclosed form a pupa cooled for 62 days; (**i**) dorsal forewing of a wild female; (**j**) dorsal forewing of a female emerged from a pupa cooled for 40 days; (**k**) ventral forewing of a wild female; (**l**) ventral forewing of a female eclosed form a pupa cooled for 40 days; (**m**) dorsal hindwing of a wild female; (**n**) dorsal hindwing of a female emerged from a pupa cooled for 40 days; (**o**) ventral hindwing of a wild female; (**p**) ventral hindwing of a female eclosed form a pupa cooled for 40 days.

We used *Polyommatus icarus* for our experiments because the males of this butterfly species have uniform blue colouration on their dorsal wing surfaces, while in Central Europe, the typical females are brown. *P. icarus* is perhaps the most widespread species of lycaenid butterfly in the Palaearctic region, from the Atlantic to the Pacific Ocean, and in Europe from Scandinavia to Sicily and Crete. The distribution of this species comprises very different climatic and biogeographical regions. Still, the typical individuals from different habitats and distant geographic regions are very similar to what is shown for the wild individuals in Fig. 1. An additional advantage is provided by the uniform blue colouration of the dorsal wing surface of the males, which enables precise measurement of any colour modification using UV-VIS spectrometry^24^. For both sexes, the ventral side of their wing surface exhibits an identical, complex pattern, which is of pigmentary origin. *P. icarus* is an ecologically plastic and abundant lepidopteran species that produces one generation in the northern limit of the range^26^ and up to three to five generations in the southern part of the range annually^27^. While the pattern variations of the ventral wing surface have been studied in detail^27, 28^, the variability of the dorsal wing surface colouration has been neglected, except for the work of Robertson^26^ focusing on the British Isles. The variations of the ventral pattern exhibited clear signs of geographical grouping^27^. It is well known that the females may exhibit a certain degree of blue colouration, which also shows geographic differences. Their frequency increases from 0.05% in northern populations to 5% in southern ones and decreases from 15% in western populations to 0.1% in the eastern part of the range^27^. In particular, in Hungary, typical females are dominantly brown; a survey of exemplars in the collections of the Hungarian Natural History Museum (more than 1000 females) showed that 10% of the females exhibited a certain, individually variable level of blue colouration on their dorsal wing surfaces.

Like most European lycaenids, *P. icarus* also adopts the strategy of overwintering as larvae. This strategy is common for 78% of the Polyommatinae in Europe^29^. Except in the northernmost part of its range, *P. icarus* has more than one brood per season^30^. The last generation produced in a season enters diapause and overwinters in the form of half-grown (second or third instar) larvae^30^. In Northern Scotland and Sweden, the flight period of imagines of *P. icarus* extends from June to September^31^, so that pupae may eventually be subjected to low temperatures for a few days before this period. Continuous and prolonged periods in which the pupae are subjected to temperatures in the range of 5 °C are unlikely. On the other hand, the species may have encountered such periods during past glaciations^32^.

## Results

### Pigment-based patterns on the ventral side of the wings

**Figure 2:**
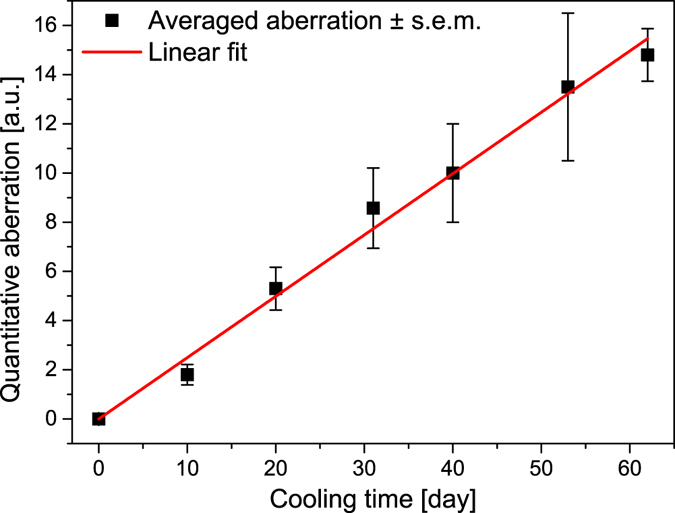
The averaged quantitative aberration of the ventral wing surfaces versus the cooling time of the pupae. For details of the conversion of pattern alteration into numerical values see the Supplementary Table 1. The red line is a linear fit to the numerically evaluated values of aberration (a = 0.24941 ± 0.00556, R^2^ = 0.99653), the error bars correspond to the standard error of the mean (s.e.m.).

The ventral side of the wing surfaces of both the males and females exhibited similar complex pigmentations (Fig. 1c,g,k and o): basal, medial and submarginal patterns comprised of black spots with white rings and orange lunules, typical for the subtribe Polyommatina. In fact, this pattern is used by taxonomists to identify these species. It is possible to assign a certain numerical value to any deviation from the standard pattern (for examples of pattern deviations, see Fig. 1d,h,l and p) observed on normal individuals, enabling the quantitative evaluation of the degree of aberration (for the details of the procedure, see Supplementary Table 1). We qualified and coded the wing size, ventral wing-surface colouration and pattern in comparison with individuals of the control group as a standard. The numerical value of the aberration was averaged for butterflies eclosed from pupae cooled for the same number of days, and the results are plotted in Fig. 2. One may observe that the quantitative value of the aberration shows a very closely monotonic and linear variation with the length of the cooling period endured by the pupae.

### Structural colour on the dorsal side of the wings

**Figure 3:**
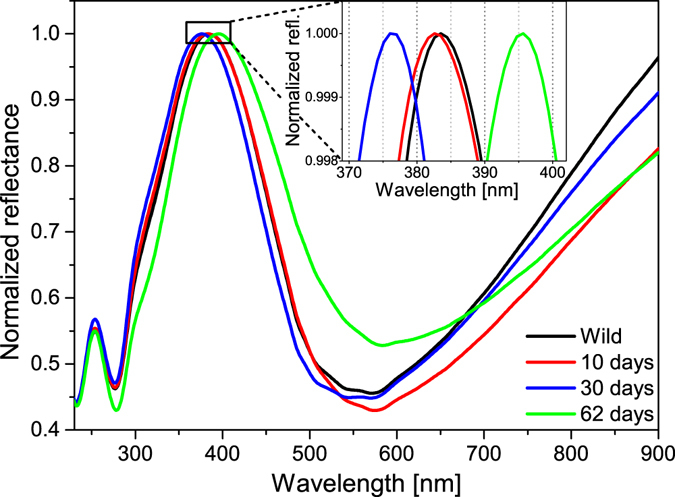
Normalized reflectance spectra averaged over all four wings of the exemplars. Wild males, and males emerged from pupa cooled for 10, 30, and 62 days respectively. The inset shows the peak region of the curves in more detail.

**Figure 4:**
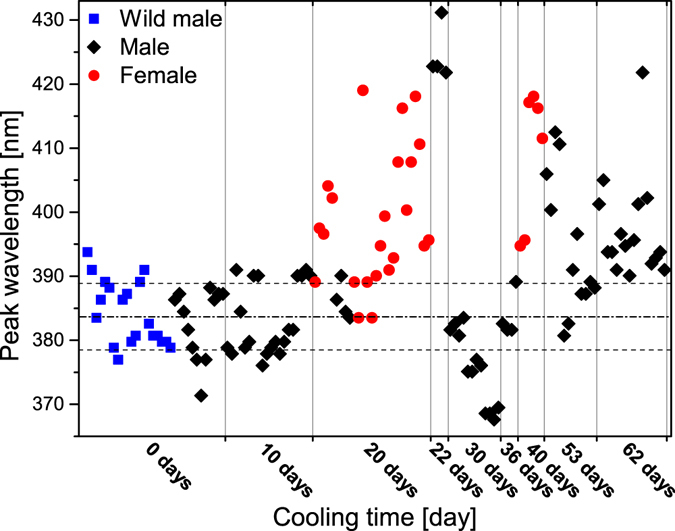
Spectral position of the blue reflectance peak for all the investigated exemplars. When possible all four wings of an individual are represented as individual measurement points close to the same vertical line. Horizontal broken lines indicate the range of wild males with centre at 384 nm marked by dotted broken line. The data of pupae cooled for a certain time are separated by thin vertical lines. We used only the wings sufficiently extended after eclosion to allow reliable spectral measurements.

Like many lycaenids, *Polyommatus icarus* exhibits sexual dichromatism; the dorsal side of the wing surface is structural blue in the case of the males^5^ and pigmentary dark brown for the females (see Fig. 1). In Hungary, the typical female is dominantly brown on the dorsal wing surface, whereas in Northern Europe, females with partial blue colouration may be found^33^. We used our earlier results from the investigation of natural variations in the blue colouration of the males within a given population of *P. icarus* ^24^ to discriminate between the natural colour variation and the effects induced by the prolonged cooling of the pupae. The reflectance of the dorsal wing surfaces (all four wings of each individual) was measured using UV-VIS spectroscopy. As typical examples, the spectra obtained from averaging the measurements on all four wings are shown in Fig. 3 for wild males and for males eclosed from pupae cooled for 10, 30 and 62 days, respectively. All spectra were normalized to the reflectance maximum to facilitate comparison. One can clearly observe that the 10 days of cooling did not result in significant alterations of the spectra. The position of the reflectance maximum in the blue region (365–432 nm) was located and plotted as a function of the cooling time for all the cooled pupae in Fig. 4. The data for the first 5 specimens (0 days of cooling) were selected randomly from a larger set of 25 specimens^24^; wild individuals were used for comparison. The next 3 sets of points correspond to reared males that were not subjected to cooling. Cooling for 10 days did not produce significant deviations in the peak position of the blue reflectance. Cooling for longer durations caused measurable deviations from the normal biological variation of the blue colour of the male *P. icarus* ^24^ and induced the appearance of blue scales on all the females, which have brown dorsal wing surfaces in the wild population. The possible cause of the shift in the spectral position of the reflectance maximum may be related to the slight alteration of the dimensions of the photonic nanoarchitecture or to changes in the scale arrangement.

### Optical microscopy of the wing scales

**Figure 5:**
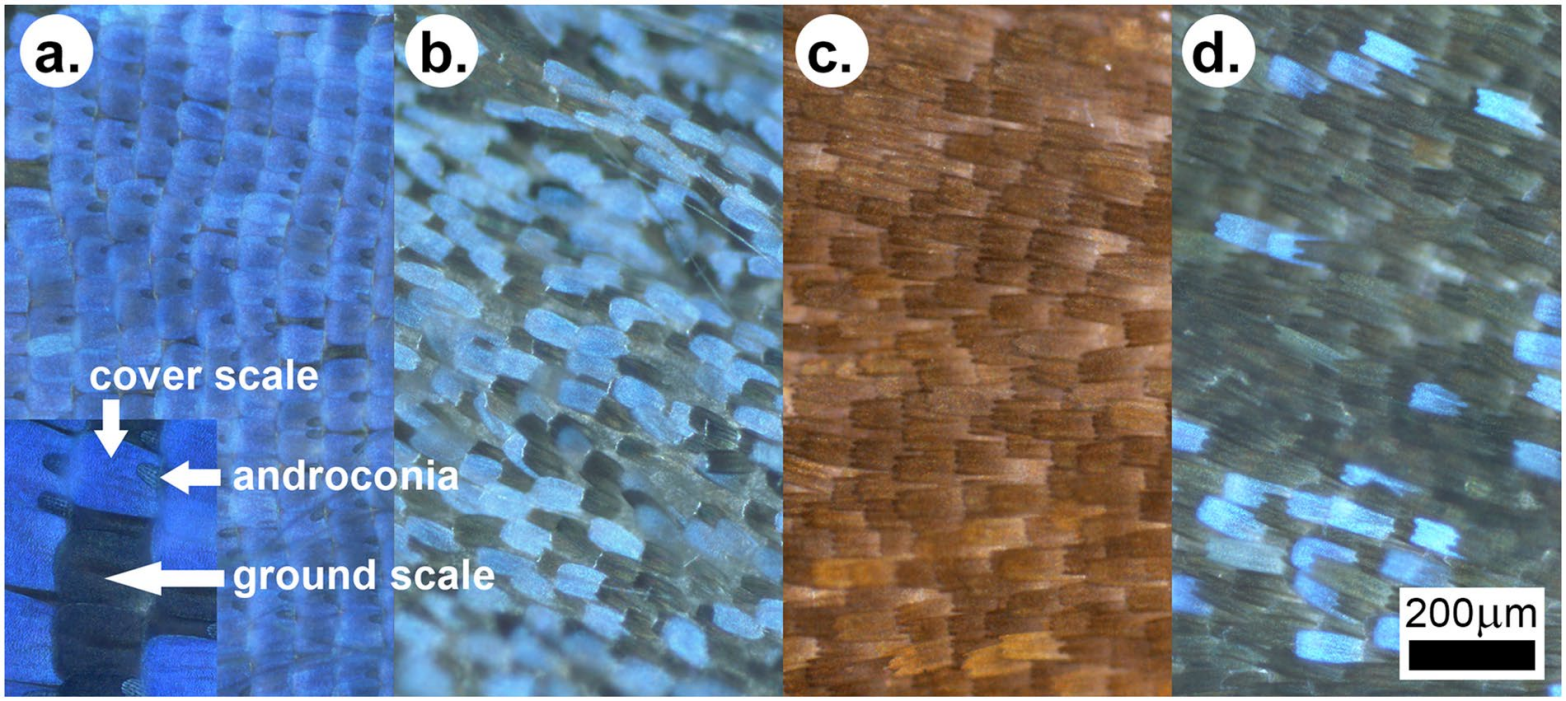
Optical microscopy of the wings. (**a**) Wild male, (**b**) male from a pupa cooled for 22 days, (**c**) wild female, (**d**) female from a pupa cooled for 20 days. The inset in the lower left corner shows magnified examples of the cover scales, ground scales and the androconia of the males.

Typically, the butterfly wing has two layers of scales: cover scales and ground scales. The latter are only partly visible under the former, as they are located between the cover scales and the wing membrane^34^. Most frequently, only the cover scales are coloured by pigments or by photonic nanoarchitectures. The ground scales usually exhibit a simple architecture composed of longitudinal ridges and crossribs and contain melanin^5, 35^. Additionally, for the scale types mentioned above, the males of *P. icarus* also possess androconia, which are special types of scales that play a role in scent production. The examination of the wings by optical microscopy revealed clear differences in the ways in which the scales were arranged on the wing surface of the cooled and reference specimens (Fig. 5). On the dorsal, blue side of the wild males (Fig. 5a), the cover scales generating the structural colour were arranged in strictly regular rows, which resembled the way in which tiles are arranged on a roof. As will be discussed in more detail later, these scales contain the photonic nanoarchitecture responsible for the blue colouration^5^. Hereafter, when mentioning blue scales, we are referring to the cover scales that contain the photonic nanoarchitectures, even in the cases when the colouration is not visible, such as in the SEM and TEM images. The rows of the blue scales were separated by rows of androconia that were also arranged in a regular manner. On the wing surface of the male that emerged from a pupa cooled for 22 days (Fig. 5b), the regular arrangement of the scales had been completely disordered. The blue scales with structural colour, the black scales (these were ground scales containing melanin) and the androconia were mixed, and no tile-like arrangement could be identified. In addition, the morphology of the blue scales exhibited variations: one could identify regular-shaped blue scales and narrow blue scales. Microscopic images characteristic of the wings of the wild females are shown in Fig. 5c. Only brown scales were visible, which clearly had different shapes (with a dentate apex vs. a round apex) compared with the blue scales on the dorsal wing side of the males. Microscopic examination revealed blue scales on the dorsal side of the wings of the females that emerged from pupae cooled for 20 days, as shown in Fig. 5d. These observations were valid for all the cooled females exhibiting scattered blue colouration on their dorsal wing surfaces.

### Scanning electron microscopy (SEM) of the wing scales

**Figure 6:**
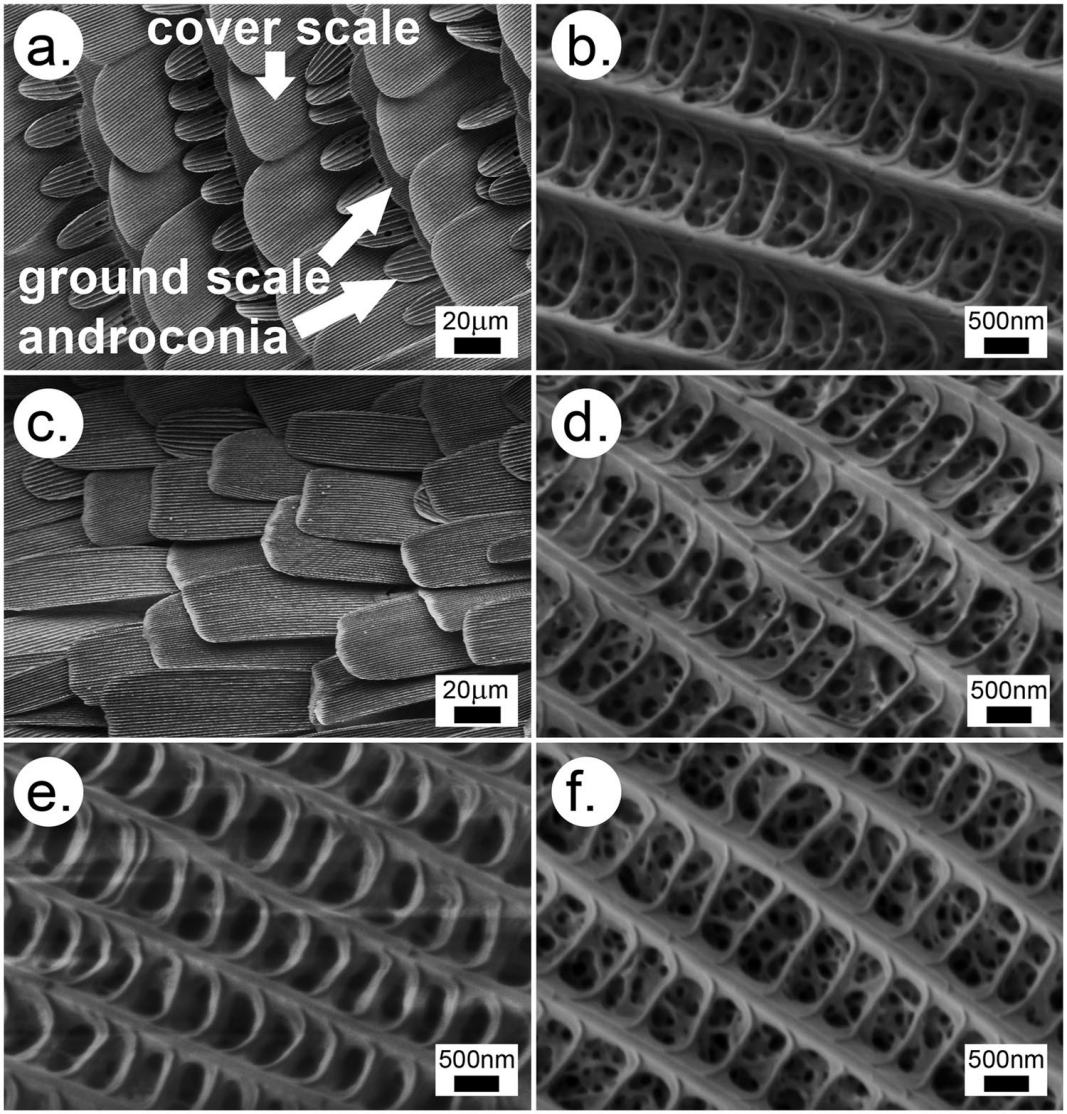
SEM images of the wing scales of the wild male and of the male eclosed from a pupa cooled for 22 days. (**a**) Wild male, overview of the regular rows of scales, the white arrows point to the cover and ground scales and to the androconia (**b**) wild male, pepper-pot structure in the volume of a blue scale, (**c**) cooled male, overview of the disordered scales, (**d**) cooled male, pepper-pot structure in the regular shape blue scale, (**e**) cooled male, black scale, (**f**) cooled male, pepper-pot structure in a narrow blue scale.

Figure 6a shows an overview of the rows of scales of a wild male, and Fig. 6b shows the structural details of the pepper-pot structure^3, 5, 36^ in the lumen of the scale. In Fig. 6a, one may remark that under each row of cover scales, there were ground scales arranged also in regular rows. In Fig. 6d and f, the SEM images of the scales of the male eclosed from a pupa cooled for 22 days are shown. Both blue scales, those of the wild male in Fig. 6d and of the cooled male in Fig. 6f, had similar pepper-pot structures, while the scales appearing as black scales in the optical microscope image in Fig. 5b had the typical structure of the ground scales, as shown in Fig. 6e.

**Figure 7:**
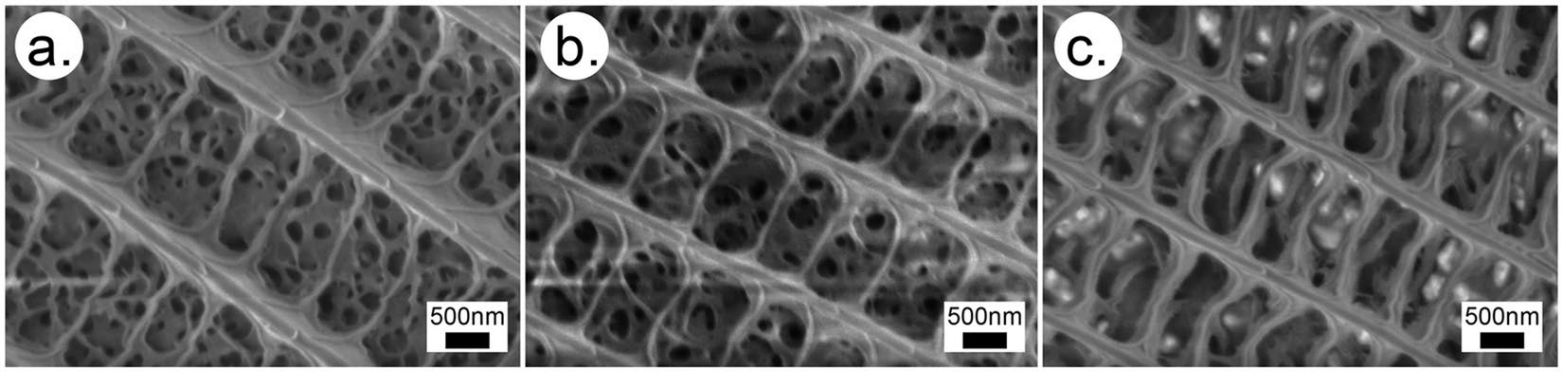
SEM images of the wing scales of the female eclosed from a pupa cooled for 20 days. (**a**) Dentate apex blue scale, (**b**) rounded apex blue scale, (**c**) brown scale.

The SEM images of the blue scales visible between the brown cover scales of the females that emerged from cooled pupae are shown in Fig. 7a and b. One may observe in the scales (through the windows formed between the longitudinal ridges and crossribs) that their lumens were filled with a similar pepper-pot-like structure. However, this structure was clearly different from that seen in Fig. 6d. The nanoarchitecture was less regular, and the holes were frequently covered by a thin layer of chitin. Figure 7c shows the structure of a regular brown scale.

### Transmission electron microscopy (TEM) of the wing scales

**Figure 8:**
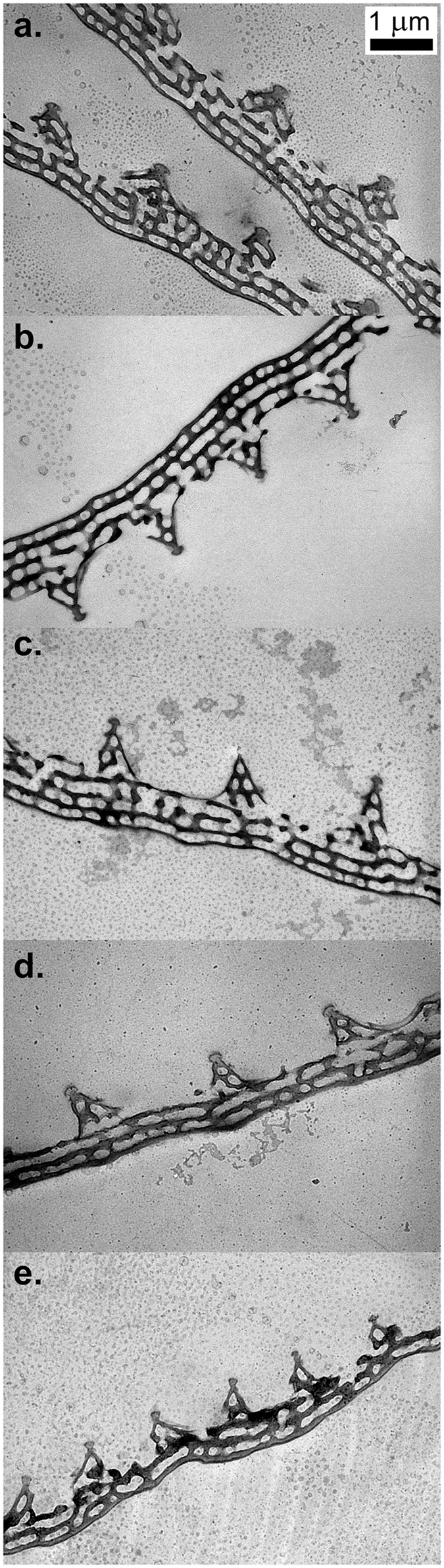
TEM images of cross sections through the blue coloured scales of males and females. (**a**) Wild male, (**b**) male emerged from a pupa cooled for 22 days, (**c**) male emerged from a pupa cooled for 62 days, (**d**) female emerged from a pupa cooled for 20 days, (**e**) female emerged from a pupa cooled for 40 days.

TEM examination of the wing cross sections revealed the inner volume nanostructure (in the plane perpendicular to that seen in the SEM images) of the wing scales. As seen in Fig. 8, the cross-sectional nanostructure of the blue cover scales of the males did not exhibit differences that could be associated with the increase in the cooling time. Characteristic differences could be observed between the scale structure of the males and that of the females; the latter had fewer layers and appeared more irregular than the scales of the males. The brown cover scales of the females had a very simple structure composed of longitudinal ridges linked by crossribs, which was very similar to the structure shown in the SEM image of Fig. 6e.

## Discussion

The most significant changes induced by the prolonged cooling of the pupae were found on the ventral wing surfaces of both sexes, in the pigment-generated patterns. As one can see from Figs 2 and 4, continuous cooling at 5 °C for 10 days induced only a minor degree of aberration in the pigment-generated pattern on the ventral wing surface and practically no change in the blue sexual signalling colour on the dorsal wing surface. This is further supported by the spectra in Fig. 3.

The magnitude of the quantified aberration of the pigment-generated pattern on the ventral wing side increased with the increase in the cooling time, as shown in Fig. 2. The increase was monotonic and at 40–60 days of cooling led to a pattern consisting of a strongly reduced number of basal, medial and marginal spots in both males and females, as shown in Fig. 1. The observed ventral wing pattern alterations were of the so-called reduction type, similar to those observed by Otaki and co-workers in a northward-expanding wild population of *Zizeeria maha*, which were later reproduced in the laboratory by an artificial cold-shock treatment^13^. Following the cold shock, three types of pattern modifications were observed: the reduction type (similar to our results shown in Fig. 1, rightmost column), the outward type (consisting of the outward elongation of black spots towards the outer wing margin), and the inward type (consisting of the elongation of black spots towards the wing base). These pattern modifications could be fixed by selective breeding^13^. It is worth mentioning here that similar-looking pattern modifications, such as the inward and outward type, were produced by selective breeding of *P. icarus* starting from a wild female called “basielongata”, i.e., the inward type. In later generations, following selective breeding, 100% of the individuals with modified wing patterns were obtained^37^.

When carefully examining the wild exemplars constituting the *P. icarus* specimens collected in the Carpathian Basin and housed in the Hungarian Natural History Museum, less than 0.1% of the males showed ventral wing pattern aberration, and most of the displayed aberrations were identical to the phenotypes of the resultant 10 day cooling experiments, i.e., approximately 10% of the magnitude of the aberrations present after 62 days of cooling. Four specimens were found that were similar to the aberrations we produced in longer cooling experiments (Supplementary Fig. 1). Some very rare wild exemplars also showed alterations in the pattern on their ventral wing surface that were comparable with those found after 62 days of cooling (Supplementary Fig. 2). Interestingly, *P. icarus* has exhibited similar pattern modifications^37^, as reported for cold-shocked *Z. maha* ^13^.

Contrary to wing pattern deviations from the standard, the changes in the blue structural colouration of the male dorsal wing surface did not increase monotonically with the increase in the cooling time (Fig. 4). The character of the changes strongly suggests that in the case of the structural colouration, the individual variations had a much stronger impact compared with the pigment-coloured wing surface. As shown by the microscopic images in Figs 5–8, the spectral changes were not highly associated with the change in the colour generating nanoarchitectures but more with the way in which the coloured wing scales were arranged on the wing membrane. While for the wild specimen (Figs 5a and 6a), the scales were arranged in neat rows; in the case of the male that was cooled for 22 days, a strongly disordered scale arrangement was already visible (Figs 5b and 6c). This disordering may cause the incident light to enter under slightly different angles on the different scales. One can see in Fig. 8 that the cross section of the scales generating the blue colour shows perforated multilayer structures, and multilayers are known to be iridescent. It was shown recently that blue-coloured *P. icarus* wings show iridescence^24^.

The females of *P. icarus* in Central Europe most frequently have a dominantly brown dorsal wing surface. On the other hand, in Northern Scotland^38^ and Southern Sweden^33^ females with a pronounced blue colouration are frequently observed. At the southern end of the range of *P. icarus*, where the climate is hot and dry, such as in Turkey^39^ and Morocco^40^, bluish females are not unusual. It was reported that larvae of *P. icarus mariscolor*, captured from a Scottish population and reared in Southern England, retain univoltine character but produce females without bright blue scaling on their dorsal wing surface, indicating the ecological control of blue suffusion^41^. Because the above observations regarding the occurrence of females with a certain degree of blue colouration on their dorsal wing surfaces include both cold and hot stress, it is realistic to assume that the stress itself (irrespective of whether it is cold or hot stress) is the cause that generates the blue colouration in females. This would explain why the blue colouration in females is frequently found on the edges of the range of *P. icarus* species. Quite remarkably, all of the females from our cooling experiments were more or less flushed with blue cover scales. As one can see in Fig. 4, both the females cooled for 20 days and the females cooled for 40 days had well defined blue maxima in their reflectance curves in the range of 385–420 nm. The blue scales are visible in the optical microscope (Fig. 5d), SEM (Fig. 7) and TEM (Fig. 8d and e) images. This is a clear indication that in our case, the thermal stress (prolonged cooling) induced the appearance of the blue scales on the dorsal wing surface of the females. The TEM images in Fig. 8 show that the scales of the females that changed towards blue colouration had fewer layers and a more disordered nanoarchitecture than the blue scales of the males. This is well supported by the SEM images, too (Fig. 7).

The average of all the butterfly spectra eclosed after 20 days of cooling (one male and 11 females) was calculated, and this average was scaled between 0 and 1 (to facilitate comparison with the normalized reflectance curves in Fig. 3). An excellent overlap was found for the spectral positions of the reflectance curves corresponding to the males cooled for 62 days (Supplementary Fig. 3). This shows that the colour generated by the nanoarchitectures produced by the pupae cooled for 20 days did not differ significantly from that produced by the pupae cooled for 62 days. This is clearly a different behaviour when compared with the changes found on the ventral wing surfaces. It also shows that despite the higher degree of disorder found in the nanoarchitectures of the blue scales of the females, the “construction plan” of the nanoarchitecture that was encoded in the genes of the males and females was essentially the same.

Clearly, the colouration generated by pigments and the colouration generated by nanoarchitectures reacted differently to the stress produced by prolonged cooling of the pupae. This finding may be less surprising if one considers that the two processes, scale formation and pigmentation, are well separated in time^25^. Scale formation is a complex process during which each scale is secreted individually by a single epithelial cell^34^. This process is not yet well understood at the nanoscale level. The blue colouration is much more stress resistant than the pigment-generated pattern of the ventral wing surface. The small magnitude of the changes induced by the prolonged cooling of the males, compared to the alteration of the ventral pattern, is well characterized in Figs 3 and 4.

The possible reasons for the occurrence of females with a variable amount of blue colouration on their dorsal wing surface in natural populations have received some attention^42^. One possibility is the advantage of more visible bluish colouration at the edges of the range, where the population density is low and the duration of the flight period is reduced, compared to the less conspicuous brown colouration. During the succession of the Quaternary glacial/post-glacial cycles, the *P. icarus* population in Europe was reduced to five refuges around the Mediterranean^32^; partially blue-coloured females may have had an advantage in these circumstances. Therefore, the prolonged period of cooling used in our experiments, which halted pupal development, may have switched on survival mechanisms of the species in the females associated with the appearance of the blue scales. As mentioned above, it cannot be excluded that thermal stress (positive or negative) switches on this survival mechanism.

The scatter in the magnitude of the peak position deviation of the blue reflectance maximum of the males and females, shown in Fig. 4, for cooling times exceeding 20 days, may be associated also with cryptic genetic variations^43, 44^, which manifest themselves only under stress conditions. Mild heat shock has been reported to induce polyphenism in larvae of the tobacco hornworm, *Manduca sexta*, by revealing hidden genetic variations due to stress^45^. For a more detailed investigation of this possibility, further experiments are needed with larger numbers of individuals subjected to cooling for longer durations.

Despite the known overwintering strategy of *P. icarus* as second or third instar larvae^29, 30^, some of the pupae were able to survive for 62 days at a temperature of 5 °C. The pupae removed from the refrigerator had the same green colouration as that observed immediately after pupation. From 5 pupae, the first butterfly eclosed after 9 days, and the last one eclosed after 12 days. The colour changes of the pupae developed as usual for the uncooled pupae (Supplementary Fig. 4). The time interval and the colour evolution in the post-cooling pupal stage appeared to indicate that under the given conditions, the pupal development was completely stopped or extremely slowed down.

Two distinct kinds of responses were identified after the prolonged cold stress: i) one occurred on the pigment-coloured ventral wing surface, the magnitude of which was proportional to the duration of cooling and, despite small individual variations, exhibited the same trend for all individuals, irrespective of their sex; and ii) the other occurred on the dorsal wing surface, which exhibited structural colouration that did not have a magnitude that was proportionally dependent on the cooling duration and exhibited a much more pronounced individual variation, possibly revealing hidden genetic variations. A possible source of these hidden genetic variations may be the history of the species during the glaciations of the Quaternary Period, when the range suitable for survival was gradually reduced to regions around the Mediterranean.

It is remarkable that for even the most extreme aberrations of the pigment-generated pattern on the ventral wing surface, it was possible to find similar-looking rare exemplars in the museum collection (see Supplementary Fig. 2). This indicates that these modifications were reproducible and may occur under extreme conditions in wild populations as well.

## Methods

### Ethics statement

The Common Blue butterfly, *Polyommatus icarus*, can be studied without any specific permission in Hungary. It is not an endangered or protected species. The stock for rearing was collected from natural habitats outside the boundaries of any protected area for nature conservation.

### The butterfly

Systematic placement: *Polyommatus icarus* (Rottemburg, 1775) (LYCAENIDAE: Polyommatinae: Polyommatini: Polyommatina).

*P. icarus* is one of the most widespread representatives of the family Lycaenidae in the Palaearctic region and is the most widely distributed species of the subtribe Polyommatina. *P. icarus* is an ecologically versatile species inhabiting open habitats including natural or secondary steppes but can also be found in both rural and urban regions. This makes the species especially suitable for environmental studies. In its wide range, there are one to five annual generations depending on the altitudinal and longitudinal circumstances. In bright sunny weather, males patrol for females suitable for mating. After copulation, females oviposit individually on living parts of the Fabaceae larval host plant. The immature larva hibernates during the 2nd or 3rd stage. The larvae are facultatively myrmecophilous; in captivity they are cannibalistic.

### Sample collection

Museum materials were used to check the appearance of aberrant specimens in natural conditions. One has to observe here that the fraction of blue females in such collections may over-represent the fraction in wild populations, as collectors tend to keep specimens that exhibit differences with respect to the common exemplars. In the populations that we used for capturing the females for our rearing experiments, we did not observe blue females in the period from 2005–2016, and none of the females used for laying eggs in our experiments exhibited blue dorsal wing surface colouration.

### Rearing

Wild females were captured and placed in groups of three, with three males in cages (Supplementary Fig. 5) suitable for oviposition. In the cages, in 20 L containers, three types of larval food plants were provided: *Trifolium repens*, *Trifolium pratense* and *Medicago sativa*. The cages, located in a garden-type environment, enabled the butterflies to survive for several weeks under close to natural conditions. The typical duration of the oviposition was two weeks. In addition to the flowering plants, the butterflies were fed a sugar water solution, which they willingly accepted. The eggs were deposited preferentially on the topmost leaves and flowers of *M. sativa* (Supplementary Fig. 6).

After the eggs hatched, the young larvae were left to feed freely on the plants provided in the cages as long as the status of the plants in the cage did not start to deteriorate dramatically. At this stage, all larvae were collected, usually during the third or fourth instar, placed individually in plastic Petri dishes and given fresh leaves of *Trifolium* every day until pupation. It was not possible to tell in the early pupal status if the individual would be male or female; this made it difficult to obtain an equilibrated distribution of the sexes between the different cooling durations. Three pupae were allowed to eclose without cooling under laboratory conditions, which are included in the reference group randomly selected from wild exemplars^24^.

### Cooling experiments

For the pupae intended for the cooling experiments, immediately after pupation, their individual Petri dishes were placed in larger sealed containers in which water was left to evaporate continuously to avoid the fatal drying of the pupae during the several weeks of cooling. No illumination was provided during cooling. After the end of the intended cooling period, the larvae were allowed to eclose in the laboratory at a temperature of 23 °C. Care was taken to provide appropriate humidity levels during this period by suspending the pupae in a net over wet sand. The eclosion time of the cooled pupae was typically in the range of 9–12 days. A succession of photos showing the daily colour changes prior to eclosion of the pupae cooled for 62 days is shown in Supplementary Fig. 4. After cooling for 10 to 20 days, all butterflies eclosed normally from the pupae; longer cooling times in the range of 40 to 50 days yielded large variations in the normal eclosion rates in the range of 30% to 60%, with approximately equal numbers of abnormal eclosions and dead pupae, which did not eclose even after several weeks of being kept in an environment with adequate temperature and humidity.

### UV-VIS Spectroscopy

To characterize the optical reflectance properties of the wings, an Avantes HS-1024X122 TEC UV-VIS fibre optic modular spectrophotometer was used. All four wings of every specimen were removed and placed as flat as possible on the integrating sphere (AvaSphere-50) sample port. To determine the reflectance spectra, we used an Avantes WS-2 diffuse white tile as a reference. The spectral data recorded were used without any further processing. The effects of prolonged cooling are presented in detail in Fig. 4. The first group was composed of wild males used for comparison and reared males that were not subjected to cooling. The next group consisted of butterflies eclosed from pupae cooled for 10 days; these were all males. When compared to the wild butterflies, no clear differences were found. By accident, in the group cooled for 20 days, all the other butterflies, which properly expanded their wings after eclosion (to allow for spectral measurements) were females, except one exemplar. All of them exhibited a certain fraction of blue scales, similar to the females in Fig. 1j and n. The only male cooled for 20 days exhibited no significant colour (spectral) deviation from the wild reference samples. A male cooled for 22 days exhibited the largest deviation of its wing colouration towards the longer wavelengths from all the cooled samples. Three males cooled for 30 days, surprisingly, showed a shift in the reflectance maxima in the opposite direction, towards the shorter wavelengths, despite the fact that the quantified aberration of their pigment-based pattern did not deviate from the linear and monotonic trend. The males cooled in the range of 53 to 62 days showed moderate shifts in the spectral reflectance maximum towards longer wavelengths, with an average of approximately 400 nm.

### Optical microscopy

The wing scales were imaged on a Zeiss Axio Imager A1 microscope.

### Scanning electron microscopy

We used an LEO 1540XB scanning electron microscope. To ensure inspection of the correct wing scale, only a few mm^2^ of the wing was cut, attached to sample holders and placed in the microscope. Although the appearance of charging effects made imaging difficult, we did not apply a metallic coating on the samples to avoid altering their intricate nanoarchitecture.

### Transmission electron microscopy

Carefully cut pieces of wing were embedded in a special resin, and 70-nm-thick slices were cut with an ultramicrotome. Slices placed on TEM microgrids were investigated with a Philips CM20 microscope. For better visibility, a contrast enhancing stain was applied.

## Electronic supplementary material

Supplementary Information
