# Supplementary material for: Changes in structural and pigmentary colours in response to cold stress in Polyommatus icarus butterflies
Source: Sci Rep. 2017 Apr 25;7:1118. doi: 10.1038/s41598-017-01273-7 (PMC5430924; doi:10.1038/s41598-017-01273-7)
Supplement: Supplementary file 1 — Supplementary Information [file 41598_2017_1273_MOESM1_ESM.pdf]

Supplementary Information

for

Changes in structural and pigmentary colours in response to cold stress in *Polyommatus icarus* butterflies

Authors: Krisztián Kertész, Gábor Piszter, Zsolt Endre Horváth, Zsolt Bálint, László Péter Biró

Supplementary Table 1. Procedure to quantify the degree of aberration on the ventral wing side of the *Polyommatus icarus* individuals

The colour and pattern aberrations of the ventral wing surfaces were investigated for all the 49 *Polyommatus icarus* specimens. The individual aberrations of the different wing areas were evaluated and the results were summarized in the table below. The elements of the table show the normal (a0, b0, etc.) and the aberrant (a1, a2, b1, etc.) marks of the ventral wing pattern which are specified in details below the table. Every aberrant mark was quantified based on the degree of the aberration. For example, when the number of spots were reduced (c1) in the basal area, the statistical weight was “1”, but when the spots completely disappeared (c2), the given weight was “2”. These values can be seen in parentheses next to the aberration marks.
The quantified values of the aberrations were averaged for every cooling period and the results were plotted Fig. 2.

treatment	cooling time (day)	sex	status	wing shape	ventral wing ground coloration	ventral forewing basal pattern	ventral forewing median pattern	ventral forewing postmedian pattern	ventral forewing marginal pattern	ventral hindwing basal pattern	ventral hindwing median pattern	ventral hindwing postmedian pattern	ventral hindwing marginal pattern
untreated	0	female	normal	a0 (0)	b0 (0)	c0 (0)	d0 (0)	e0 (0)	f0 (0)	g0 (0)	h0 (0)	i0 (0)	j0 (0)
untreated	0	female	aberrant	a0 (0)	b0 (0)	c0 (0)	d0 (0)	e0 (0)	f0 (0)	g1 (1)	h0 (0)	i0 (0)	j0 (0)
untreated	0	male	normal	a0 (0)	b0 (0)	c0 (0)	d0 (0)	e0 (0)	f0 (0)	g0 (0)	h0 (0)	i0 (0)	j0 (0)
untreated	0	female	normal	a0 (0)	b0 (0)	c0 (0)	d0 (0)	e0 (0)	f0 (0)	g0 (0)	h0 (0)	i0 (0)	j0 (0)
untreated	0	male	normal	a0 (0)	b0 (0)	c0 (0)	d0 (0)	e0 (0)	f0 (0)	g0 (0)	h0 (0)	i0 (0)	j0 (0)
untreated	0	female	normal	a0 (0)	b0 (0)	c0 (0)	d0 (0)	e0 (0)	f0 (0)	g0 (0)	h0 (0)	i0 (0)	j0 (0)
untreated	0	male	normal	a0 (0)	b0 (0)	c0 (0)	d0 (0)	e0 (0)	f0 (0)	g0 (0)	h0 (0)	i0 (0)	j0 (0)
untreated	0	male	normal	a0 (0)	b0 (0)	c0 (0)	d0 (0)	e0 (0)	f0 (0)	g0 (0)	h0 (0)	i0 (0)	j0 (0)
untreated	0	female	normal	a0 (0)	b0 (0)	c0 (0)	d0 (0)	e0 (0)	f0 (0)	g0 (0)	h0 (0)	i0 (0)	j0 (0)
untreated	0	male	normal	a0 (0)	b0 (0)	c0 (0)	d0 (0)	e0 (0)	f0 (0)	g0 (0)	h0 (0)	i0 (0)	j0 (0)
cooled	10	male	aberrant	a1 (1)	b0 (0)	c3 (2)	d0 (0)	e0 (0)	f0 (0)	g0 (0)	h0 (0)	i0 (0)	j0 (0)
cooled	10	female	aberrant	a1 (1)	b0 (0)	c3 (2)	d0 (0)	e0 (0)	f0 (0)	g0 (0)	h0 (0)	i0 (0)	j0 (0)
cooled	10	male	aberrant	a1 (1)	b0 (0)	c3 (2)	d0 (0)	e0 (0)	f0 (0)	g0 (0)	h0 (0)	i0 (0)	j0 (0)
cooled	10	male	aberrant	a0 (0)	b0 (0)	c0 (0)	d0 (0)	e0 (0)	f0 (0)	g0 (0)	h0 (0)	i0 (0)	j0 (0)
cooled	10	female	aberrant	a0 (0)	b0 (0)	c0 (0)	d0 (0)	e0 (0)	f0 (0)	g0 (0)	h0 (0)	i0 (0)	j0 (0)
cooled	10	female	aberrant	a0 (0)	b0 (0)	c3 (2)	d0 (0)	e0 (0)	f0 (0)	g0 (0)	h0 (0)	i0 (0)	j0 (0)

cooled	10	male	aberrant	a0 (0)	b0 (0)	c3 (2)	d0 (0)	e0 (0)	f0 (0)	g0 (0)	h0 (0)	i0 (0)	j0 (0)
cooled	10	female	aberrant	a0 (0)	b0 (0)	c3 (2)	d0 (0)	e0 (0)	f0 (0)	g0 (0)	h0 (0)	i0 (0)	j0 (0)
cooled	10	female	normal	a0 (0)	b0 (0)	c0 (0)	d0 (0)	e0 (0)	f0 (0)	g0 (0)	h0 (0)	i0 (0)	j0 (0)
cooled	10	male	aberrant	a1 (1)	b0 (0)	c3 (2)	d0 (0)	e0 (0)	f0 (0)	g0 (0)	h0 (0)	i0 (0)	j0 (0)
cooled	20	female	aberrant	a0 (0)	b0 (0)	c0 (0)	d0 (0)	e0 (0)	f0 (0)	g0 (0)	h0 (0)	i1 (1)	j0 (0)
cooled	20	female	aberrant	a0 (0)	b0 (0)	c2 (2)	d0 (0)	e0 (0)	f0 (0)	g1 (1)	h0 (0)	i1 (1)	j0 (0)
cooled	20	male	aberrant	a0 (0)	b0 (0)	c2 (2)	d3 (2)	e3 (2)	f1 (1)	g3 (2)	h0 (0)	i1 (1)	j0 (0)
cooled	20	female	aberrant	a1 (1)	b1 (1)	c0 (0)	d3 (2)	e3 (2)	f0 (0)	g0 (0)	h0 (0)	i1 (1)	j0 (0)
cooled	20	female	aberrant	a0 (0)	b0 (0)	c0 (0)	d3 (2)	e0 (0)	f0 (0)	g0 (0)	h0 (0)	i0 (0)	j0 (0)
cooled	20	female	aberrant	a1 (1)	b0 (0)	c2 (2)	d0 (0)	e0 (0)	f0 (0)	g0 (0)	h0 (0)	i1 (1)	j0 (0)
cooled	20	female	aberrant	a1 (1)	b0 (0)	c0 (0)	d3 (2)	e3 (2)	f0 (0)	g0 (0)	h0 (0)	i0 (0)	j0 (0)
cooled	20	female	aberrant	a0 (0)	b0 (0)	c2 (2)	d0 (0)	e0 (0)	f0 (0)	g3 (2)	h0 (0)	i1 (1)	j0 (0)
cooled	20	female	aberrant	a0 (0)	b0 (0)	c2 (2)	d0 (0)	e0 (0)	f0 (0)	g3 (2)	h1 (1)	i2 (2)	j0 (0)
cooled	20	female	aberrant	a0 (0)	b0 (0)	c2 (2)	d3 (2)	e3 (2)	f0 (0)	g0 (0)	h1 (1)	i1 (1)	j0 (0)
cooled	22	male	aberrant	a1 (1)	b1 (1)	c1 (1)	d1 (1)	e1 (1)	f0 (0)	g1 (1)	h1 (1)	i1 (1)	j0 (0)
cooled	30	female	normal	a0 (0)	b0 (0)	c0 (0)	d0 (0)	e0 (0)	f0 (0)	g0 (0)	h0 (0)	i0 (0)	j0 (0)
cooled	30	male	aberrant	a0 (0)	b0 (0)	c2 (2)	d2 (2)	e1 (1)	f1 (1)	g2 (2)	h1 (1)	i1 (1)	j0 (0)
cooled	30	male	aberrant	a0 (0)	b0 (0)	c1 (1)	d1 (1)	e0 (0)	f0 (0)	g2 (2)	h2 (2)	i0 (0)	j0 (0)
cooled	30	male	aberrant	a1 (1)	b1 (1)	c3 (2)	d3 (2)	e3 (2)	f1 (1)	g1 (1)	h1 (1)	i1 (1)	j0 (0)
cooled	30	female	aberrant	a0 (0)	b0 (0)	c2 (2)	d1 (1)	e0 (0)	f0 (0)	g2 (2)	h2 (2)	i2 (2)	j0 (0)
cooled	30	female	aberrant	a1 (1)	b0 (0)	c2 (2)	d1 (1)	e2 (2)	f0 (0)	g2 (2)	h2 (2)	i2 (2)	j0 (0)
cooled	30	female	aberrant	a0 (0)	b1 (1)	c2 (2)	d1 (1)	e1 (1)	f0 (0)	g2 (2)	h2 (2)	i2 (2)	j0 (0)
cooled	36	male	aberrant	a1 (1)	b1 (1)	c2 (2)	d2 (2)	e2 (2)	f1 (1)	g2 (2)	h2 (2)	i2 (2)	j0 (0)
cooled	40	female	aberrant	a0 (0)	b0 (0)	c2 (2)	d2 (2)	e3 (2)	f0 (0)	g0 (0)	h0 (0)	i2 (2)	j0 (0)
cooled	40	female	aberrant	a0 (0)	b1 (1)	c2 (2)	d2 (2)	e2 (2)	f0 (0)	g1 (1)	h2 (2)	i2 (2)	j0 (0)
cooled	53	male	aberrant	a1 (1)	b0 (0)	c1 (1)	d0 (0)	e0 (0)	f0 (0)	g0 (0)	h0 (0)	i0 (0)	j0 (0)
cooled	53	male	aberrant	a1 (1)	b1 (1)	c1 (1)	d1 (1)	e1 (1)	f0 (0)	g1 (1)	h1 (1)	i1 (1)	j0 (0)
cooled	54	male	aberrant	a1 (1)	b2 (1)	c2 (2)	d2 (2)	e2 (2)	f2 (2)	g2 (2)	h2 (2)	i2 (2)	j1 (1)
cooled	62	male	aberrant	a1 (1)	b2 (1)	c2 (2)	d2 (2)	e2 (2)	f2 (2)	g2 (2)	h2 (2)	i2 (2)	j0 (0)
cooled	62	male	aberrant	a1 (1)	b0 (0)	c2 (2)	d2 (2)	e2 (2)	f2 (2)	g2 (2)	h2 (2)	i2 (2)	j1 (1)
cooled	62	female	aberrant	a1 (1)	b1 (1)	c2 (2)	d2 (2)	e2 (2)	f0 (0)	g2 (2)	h2 (2)	i2 (2)	j0 (0)
cooled	62	male	aberrant	a1 (1)	b2 (1)	c2 (2)	d2 (2)	e2 (2)	f2 (2)	g2 (2)	h2 (2)	i2 (2)	j1 (1)
cooled	62	male	aberrant	a1 (1)	b2 (1)	c2 (2)	d2 (2)	e1 (1)	f1 (1)	g1 (1)	h1 (1)	i1 (1)	j0 (0)

Wing shape: a0 = normal, a1 = deformed (smaller, etc.)

Ventral wing ground coloration: b0 = normal; b1 = darker; b2 = lighter

Ventral forewing basal pattern: c0 = normal; c1 = spots vestigial or/and reduced in number; c2 = no spots; c3 = spots larger (coalescent)

Ventral forewing median pattern: d0 = normal; d1 = spots vestigial or/and reduced in number; d2 = no spots; d3 = spots larger

Ventral forewing postmedian pattern: e0 = normal; e1 = spots vestigial or/and reduced in number; e2 = no spots, e3 = spots larger

Ventral forewing marginal pattern: f0 = normal; f1 = no orange lunules; f2 = no orange lunules, black pattern reduced or deficient

Ventral hindwing basal pattern: g0 = normal; g1 = spots vestigial and/or reduced in number; g2 = no spots; g3 = blue suffusion extended (spots covered)

Ventral hindwing median pattern: h0 = normal; h1 = spots vestigial and/or reduced in number; h2 = no spots; h3 = spots larger

Ventral hindwing postmedian pattern: i0 = normal; i1 = spots vestigial and/or reduced in number; i2 = no spots

Ventral hindwing marginal pattern: j0 = normal; j1 = no orange lunules; j2 = no orange lunules, black pattern reduced or deficient

Aberrant *Polyommatus icarus* specimens found in nature

One can find a male with similar ventral pattern to the males emerged from pupae cooled for 62 days: labelled “obsoleta” (Specimen number: 501407) and two other exemplars, identified as aberrations (Specimen numbers: 501409 and 501410) in the Cockayne collection of Natural History Museum London [1]. A fourth similarly looking exemplar can be found in the Werner Kraus collection of the Musée National d'Histoire Naturelle in Luxembourg [2] (Fig. S2).

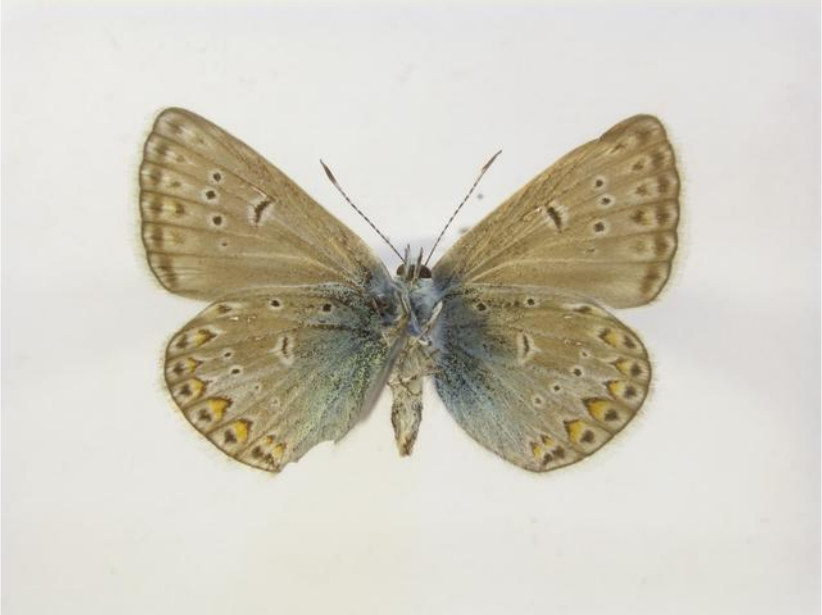

Polyommatus icarus male, collected in Kunpeszér (Hungary), 5. VI. 1998, leg. A. Máté

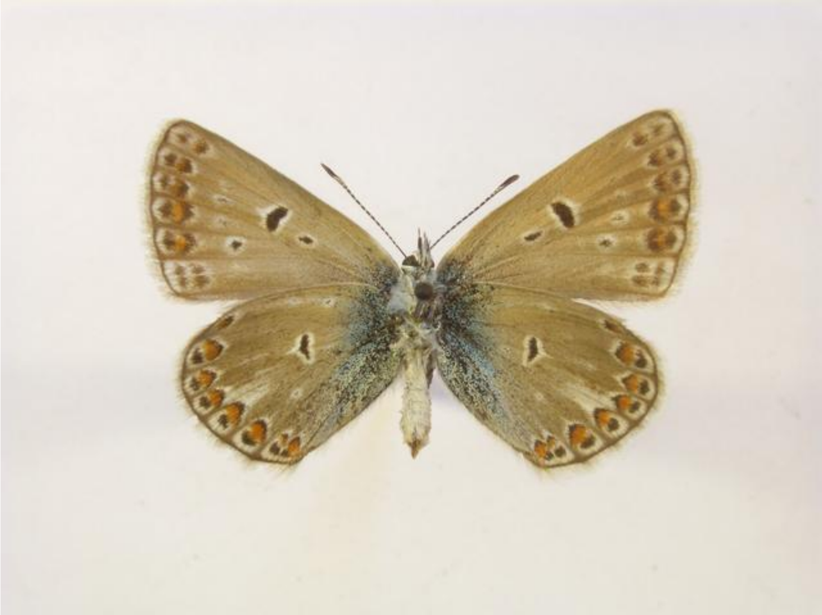

Polyommatus icarus female, collected in Lovrin (=Banat, Romania), 20. V. 1937, leg. B. Lipthay

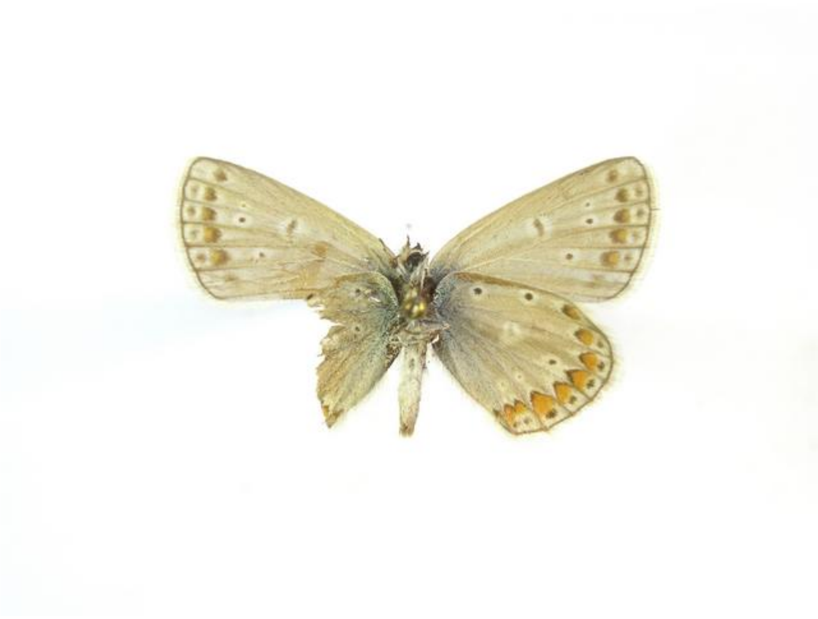

Polyommatus icarus male, collected in Monyoró (=Mânerău, Criș-region, Romania), VIII. 1991, leg. L. Diószeghy

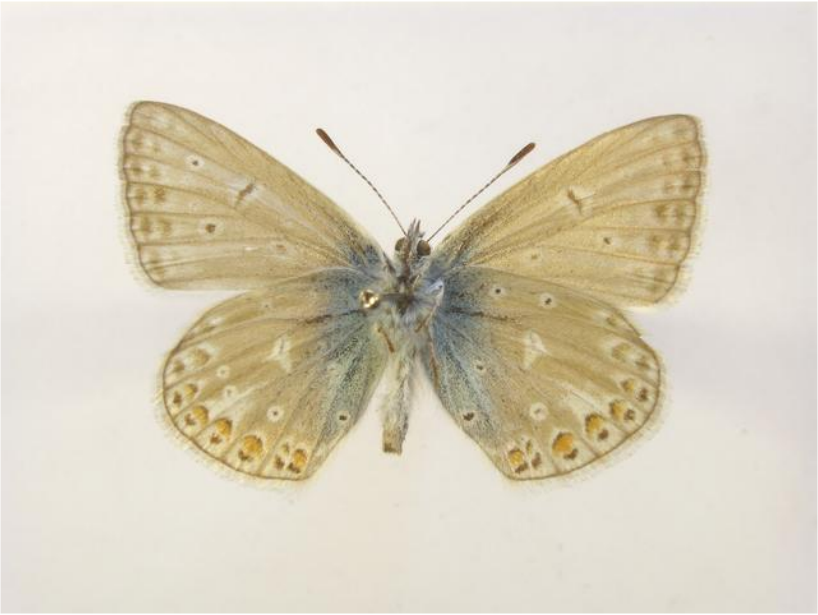

Polyommatus icarus male, collected in Eperjes (=Prešov, NE Slovakia), 8.VI. 1896, leg. Gy. Dahlström

Supplementary Figure 1: Four cases for aberrations resembling individuals eclosed after cooling exceeding 20 days, present in the collections of the Hungarian Natural History Museum, showing ventral wing surfaces (all photos: Gergely Katona, HNHM).

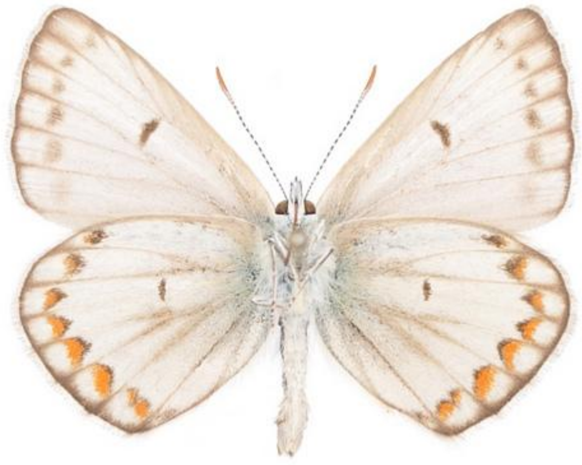

Supplementary Figure 2: An extremely rare aberrant *Polyommatus icarus* male specimen, collected in Loano (Ligurie, Italy, 9. IX. 1964, leg. W. Kraus), deposited in Musée National d'Histoire Naturelle in Luxembourg (Access was granted by courtesy of Mr. Jean-Michel Guinet; Photo by Claude Sinner).

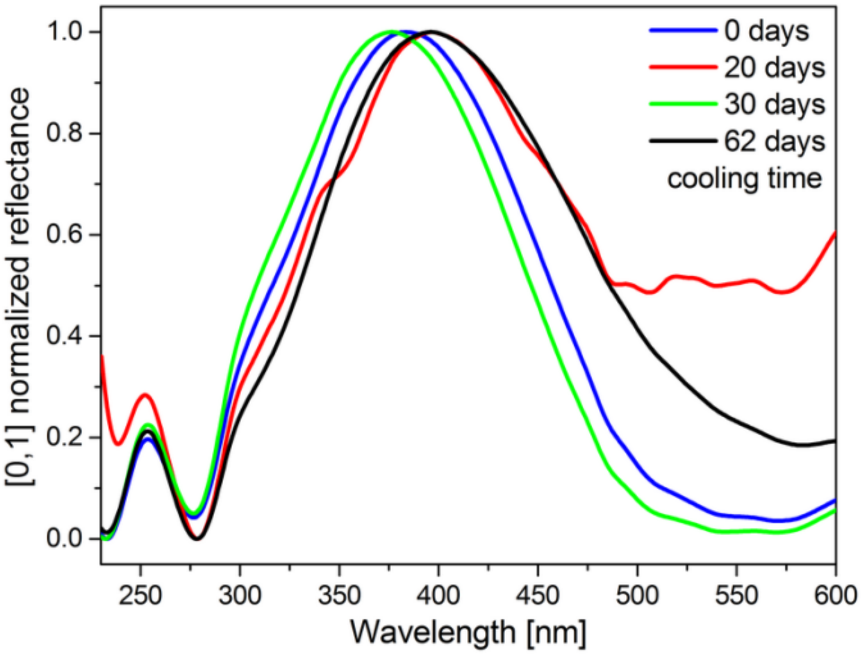

Supplementary Figure 3: Spectral position of the averaged reflectance curve for all specimens resulted after 20 days of cooling compared with the spectra corresponding to the average of wild males and males cooled for 10 and for 62 days.

Only those females were used on which it was possible to locate a clear reflectance maximum in the blue region. The reflectance measurements were carried out with an integrating sphere with a sample port of 5 mm in diameter and not all the females possessed a sufficient number of blue scales over this surface to make possible the unambiguous location of the reflectance maximum.

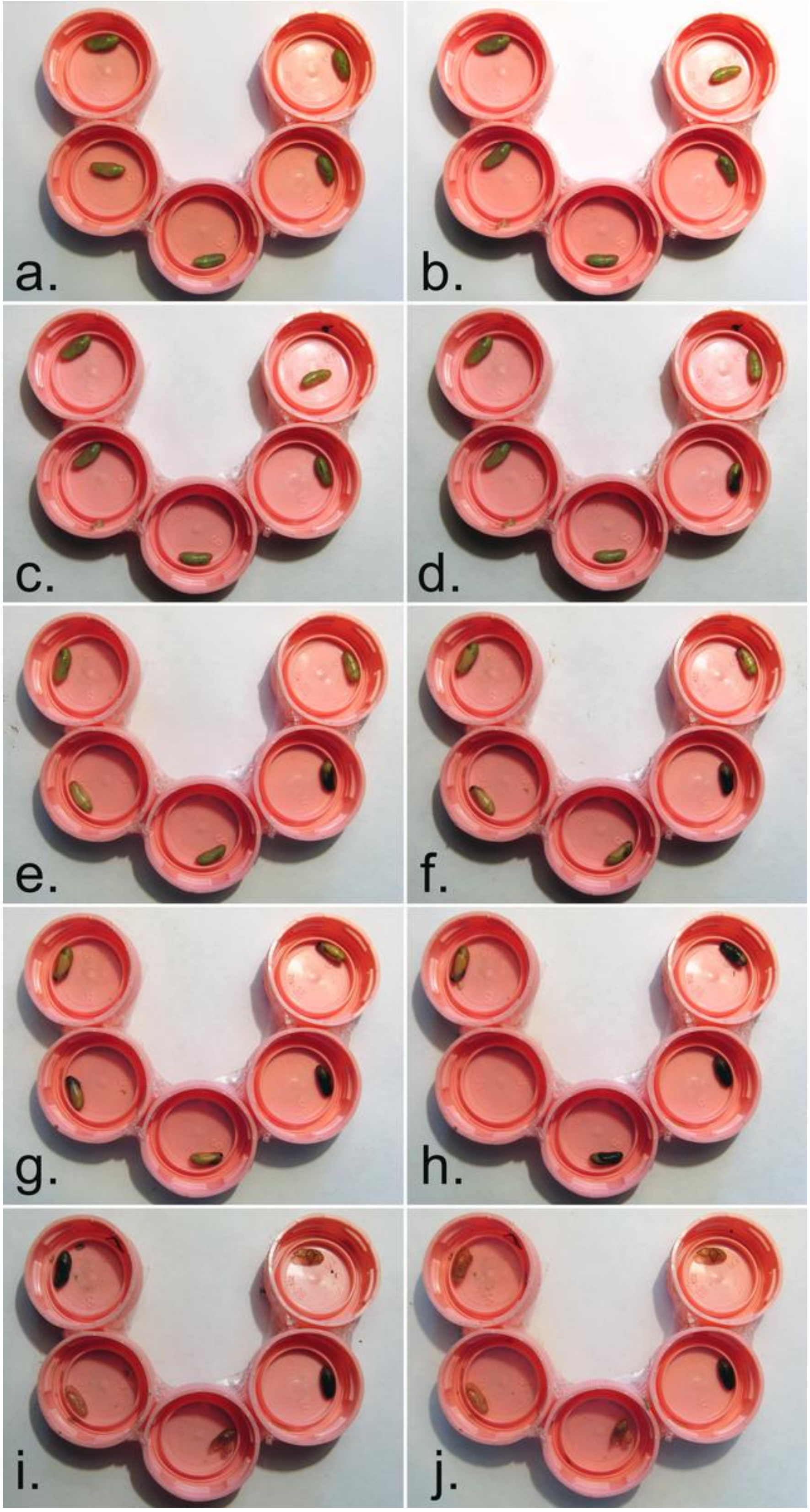

Supplementary Figure 4: Colour change of the pupae cooled for 62 days, when allowed to eclose in the laboratory at 23°C. Date of photo: (a) 2014.10.20, (b) 2014.10.21, (c) 2014.10.22, (d) 2014.10.23, (e) 2014.10.25, (f) 2014.10.27, (g) 2014.10.28, (h) 2014.10.29, (i) 2014.10.30, (j) 2014.10.31.

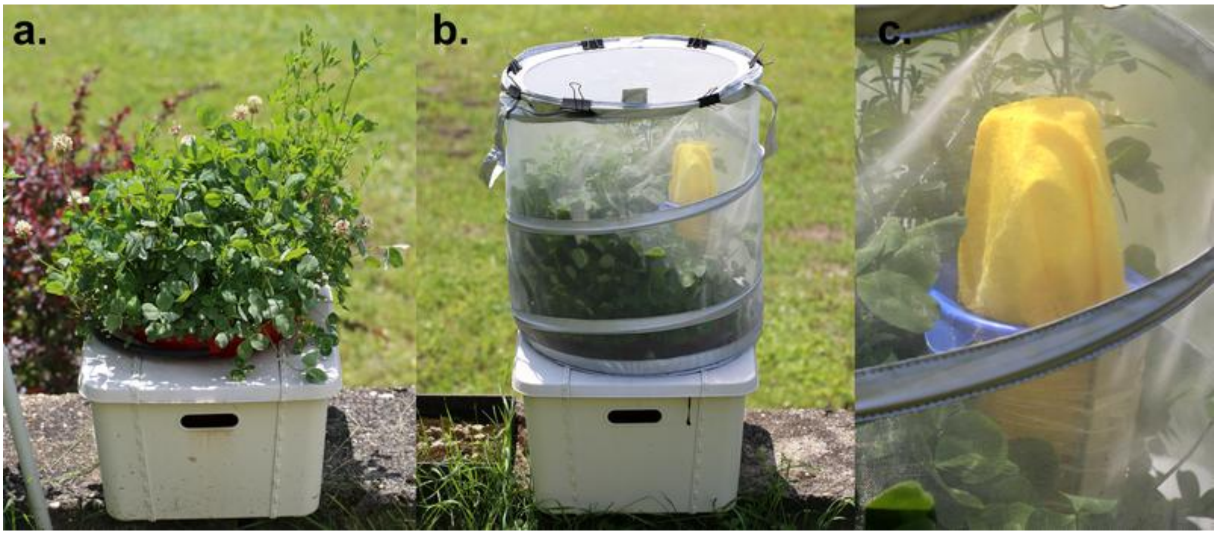

Supplementary Figure 5: Cage system used for oviposition and initial rearing of the young larvae. (a) container with the food plants prepared for receiving the females for oviposition, (b) the net and the sugar water feeder for butterflies assembled, (c) detail of the sugar water feeder.

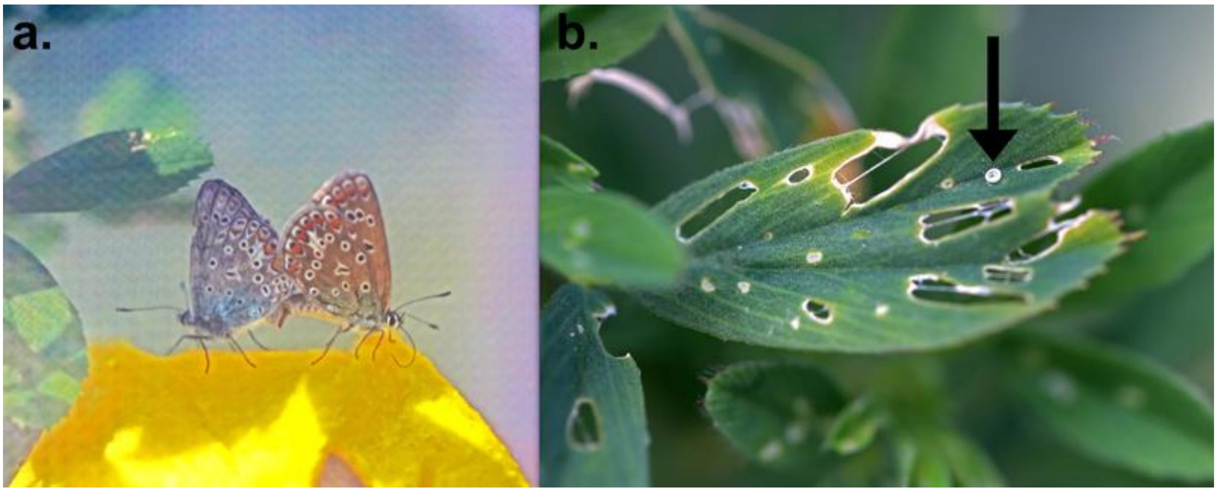

Supplementary Figure 6: (a) male and female individuals mating on the sugar water feeder, (b) hatched egg indicated by black arrow and traces of feeding left by the young larvae on a leave of *Medicago sativa*.

---

[1] http://www.nhm.ac.uk/our-science/data/british-butterflies-moths/database/taxon.dsml?option=typicalbutterflies&beginIndex=10&searchPageURL=index.dsml%3Foption%3Dtypicalbutterflies&listPageURL=browse.dsml%3Foption%3Dtypicalbutterflies

Specimen number: 501407 Type status: Sex: Male Location: Hunts. England, Collector: H.A. Leeds, Collection date: 7.6.1946 Comments: Bradley number: 1574 European checklist

number: 7163 - See more at: http://www.nhm.ac.uk/our-science/data/british-butterflies-moths/database/specimen.dsml?specimenID=10097&filename=501407X&taxonID=8973&option=typicalbutterflies#sthash.3qY5Pav2.dpuf

Specimen number: 501410, Type status: Sex: Male, Location: Salisbury England, Collector: H. Haynes, Collection date: 1933 Comments: Bradley number: 1574 European checklist number: 7163- See more at: http://www.nhm.ac.uk/our-science/data/british-butterflies-moths/database/specimen.dsml?specimenID=10100&filename=501410X&taxonID=9482&option=typicalbutterflies#sthash.ReB4lQgP.dpuf

Specimen number: 501409, Type status: Sex: Male, Location: Corfe England, Collector: Collection date: 5.8.47, Comments: Bradley number: 1574, European checklist number: 7163- See more at: http://www.nhm.ac.uk/our-science/data/british-butterflies-moths/database/specimen.dsml?specimenID=10099&filename=501409X&taxonID=9481&option=typicalbutterflies#sthash.3V8DA4p4.dpuf

[2] Personal communication and photo from Claude Sinner.
